# Emotional Contagion From Humans to Dogs Is Facilitated by Duration of Ownership

**DOI:** 10.3389/fpsyg.2019.01678

**Published:** 2019-07-19

**Authors:** Maki Katayama, Takatomi Kubo, Toshitaka Yamakawa, Koichi Fujiwara, Kensaku Nomoto, Kazushi Ikeda, Kazutaka Mogi, Miho Nagasawa, Takefumi Kikusui

**Affiliations:** ^1^Department of Animal Science and Biotechnology, Azabu University, Sagamihara, Japan; ^2^Division of Information Science, Graduate School of Science and Technology, Nara Institute of Science and Technology, Ikoma, Japan; ^3^Research Promotion Division, Department of Computer Science and Electrical Engineering, Faculty of Engineering, Kumamoto University, Kumamoto, Japan; ^4^Human Systems Laboratory, Department of Systems Science, Graduate School of Informatics, Kyoto University, Kyoto, Japan

**Keywords:** emotional contagion, dog, human, heart rate variability, environment sharing

## Abstract

Emotional contagion is a primitive form of empathy that does not need higher psychological functions. Recent studies reported that emotional contagion exists not only between humans but also among various animal species. The dog (*Canis familiaris*) is a unique animal and the oldest domesticated species. Dogs have coexisted with humans for more than 30,000 years and are woven into human society as partners bonding with humans. Dogs have acquired human-like communication skills and, likely as a result of the domestication process, the ability to read human emotions; therefore, it is feasible that there may be emotional contagion between human and dogs. However, the higher time-resolution of measurement of emotional contagion between them is yet to be conducted. We assessed the emotional reactions of dogs and humans by heart rate variability (HRV), which reflects emotion, under a psychological stress condition on the owners. The correlation coefficients of heart beat (R-R) intervals (RRI), the standard deviations of all RR intervals (SDNN), and the square root of the mean of the sum of the square of differences between adjacent RR intervals (RMSSD) between dogs and owners were positively correlated with the duration of dog ownership. Dogs’ sex also influenced the correlation coefficients of the RRI, SDNN, and RMSSD in the control condition; female showed stronger values. These results suggest that emotional contagion from owner to dog can occur especially in females and the time sharing the same environment is the key factor in inducing the efficacy of emotional contagion.

## Introduction

Emotional contagion is a primitive form of empathy and does not need higher psychological functions such as theory-of-mind and perspective taking (de Waal, 2009). Recent studies reported that positive and negative emotional contagion exist not only between humans but also among various animal species. For example, mice observe the pain behavior of other mice; an observer mouse showed evidence of contagious behavior, such as increase in freezing or pain behavior (Langford et al., 2006; Jeon et al., 2010). In chickens, mother birds showed specific responses to their chicks’ distress (Edgar et al., 2011). Therefore, emotional contagion is a fundamental function in animals that live in groups.

One important aspect of emotional contagion is that its efficacy is modulated by the relationship within a dyad. If a pair forms an affiliative relationship, such as that between siblings or the mother–infant relationship, the efficacy of emotional contagion increases (Langford et al., 2006; Jeon et al., 2010). In the theoretical model proposed by Ohtsuki et al., emotional contagion can evolve by sharing an environment for a long period; moreover, the effect of genetic relatedness is not relatively effective for the evolution of emotional contagion (Nakahashi and Ohtsuki, 2018). In fact, co-housing for more than 3–4 weeks were needed to show emotional pain contagion in mice dyads (Langford et al., 2006; Jeon et al., 2010). However, this theoretical hypothesis has not been well documented using animal models. One example was a rat experiment that did not provide direct evidence for emotional contagion but did show that pair-housed rats exhibited helping behavior after co-housing more than 2 weeks. In this example, sharing the same environment was an important factor in inducing helping behavior even the partner rat was genetically different strains (Ben-Ami Bartal et al., 2011; Bartal et al., 2014). In this previous study, the partner rats from a different strain is still genetically similar, because the rats belong to the same species and were domesticated in the laboratory conditions (Ben-Ami Bartal et al., 2011; Bartal et al., 2014). To test the hypothesis proposed by Ohtsuki (Nakahashi and Ohtsuki, 2018) and the characteristic of the emotional contagion of individual relationship, the owner–dog (*Canis familiaris*) dyad is a preferable model for evaluating the factors involved in an emotional contagion that has been established for three reasons. First, dog and owner can form a biological bond that is mediated by the bonding hormone oxytocin (Nagasawa et al., 2015). Second, relationship factors between owner and dog such as the duration of ownership (sharing the same environment) can vary, enabling a correlation analysis. Third, there is no genetic relatedness between dogs and owners.

Dogs have been reported to show behavioral or physiological contagion with humans (Jones and Josephs, 2006; Romero et al., 2013, 2014; Yong and Ruffman, 2014). One example is that dogs showed yawning contagion with humans, particularly with their owners (Romero et al., 2013). Sümegi et al. (2014) examined the presence of emotional contagion among dogs and owners and tested whether dogs show some signs of taking on their owner’s current affective state. The results suggested that the owner’s state of anxiety was contagious to their dog and the emotional contagion could be tracked by measuring changes in the dog’s memory performance (Sümegi et al., 2014).

Other studies tested dogs’ reactions to human crying. Yong and Ruffman evaluated dog’s responses to human crying, which was found elicited an increase in cortisol levels both in dogs and humans, together with submissive and alerting behavior in dogs (Yong and Ruffman, 2014). The owner also showed crying behavior in the presence of the dog. In this situation, dogs looked at and approached their owner and engaged in licking and nuzzling behavior toward the owner. These results indicated both that human crying can transmit the human emotional valence to the dogs, and that dogs can recognize and react to human emotional changes with an increased stress response.

One explanation for the dogs’ behavior toward distressed owners is that dogs feel negative emotion and so seek comfort or relief from distress (Nagasawa et al., 2009). However, it is possible that dogs may have previously received positive reinforcement for approaching crying individuals. A pet dog who approaches a distressed human family member is likely to be positively reinforced by receiving affection. To solve this concern, not only behavior but also emotion-related physiological changes both in the owner and dogs have to be assessed, and correlation analysis needs to be conducted. As mentioned above, Yong and Ruffman (2014) evaluated cortisol changes in owner and dogs; however, the emotions can changed seconds to minutes, and so a higher time resolution is needed to evaluate the accuracy of a dog’s response to the owner’s emotional status.

Heart beat intervals (R-R intervals, RRI) are not stable but contain fluctuations. Heart rate is controlled by both the sympathetic and parasympathetic nerve systems, and the balance of these two systems can determine the RRI. RRIs contain variability, not constancy, and, therefore, some indices are used as parameters for heart rate variability (HRV). The root mean square of the successive differences in RRI (RMSSD) reflects the beat-to-beat variance in HR and is the primary time-domain measure used to estimate the vagally mediated changes reflected in HRV. In contrast, both the sympathetic and the parasympathetic nerve systems contribute to the mean of the standard deviations of RRI (SDNN). Therefore, the parameters of HRV are useful indicators when measuring the autonomic nerve system activity that is caused by emotional state (Von Borell et al., 2007; Kreibig, 2010). Various studies exist that investigate the association of HRV with negative emotion (Von Borell et al., 2007). Other studies that indicate the relationship positive emotional state are also associated with HRV (Brosschot and Thayer, 2003; Katayama et al., 2016). We observed that the influence of the emotional change in dogs can be detected in HR and HRV (Katayama et al., 2016). In particular, negative and positive emotions are associated with a decline of RMSSD and a decline of the SDNN, respectively. These data indicate that RRI and HRV parameters are useful to understand the balance of the sympathetic and parasympathetic nerve systems.

In the present study, we used HRV as an index of emotional status that changes in seconds, and compared the changes of HRV indices between the owner and the dog in a stressful situation. We included the factors that can affect the efficacy of emotional contagion, such as the duration of the ownership, attachment behavior (index of a strength of bonding), for analysis. We hypothesized that emotional contagion can be observed in owner–dog dyads and showed high bonding behavior and long-term sharing within the same environment.

## Materials and Methods

### Subjects

The experiment was carried out on 34 small-to-large-sized dogs and their owners, who were recruited at animal hospitals and parks (Supplementary Table S1). We asked the owners about their health condition and that of their dogs before starting the experiment. Dogs with behavioral or physical problems were excluded. Dog’s information (age, sex, nurturing, breeds, the duration of the ownership, and daily duration of time spent with dogs (Score 1, less than 6 h: Score 2, 6 h or more and less than 9 h: Score 3, 9 h or more and less than 12 h: Score 4, 12 h or more) were obtained from the owner. All experimental procedures were approved by the Animal Ethics Committee of Azabu University (#180410-1) and the Ethical Committee for Medical and Health Research Involving Human Subjects of Azabu University (#052).

### Apparatus

**FIGURE 1:**
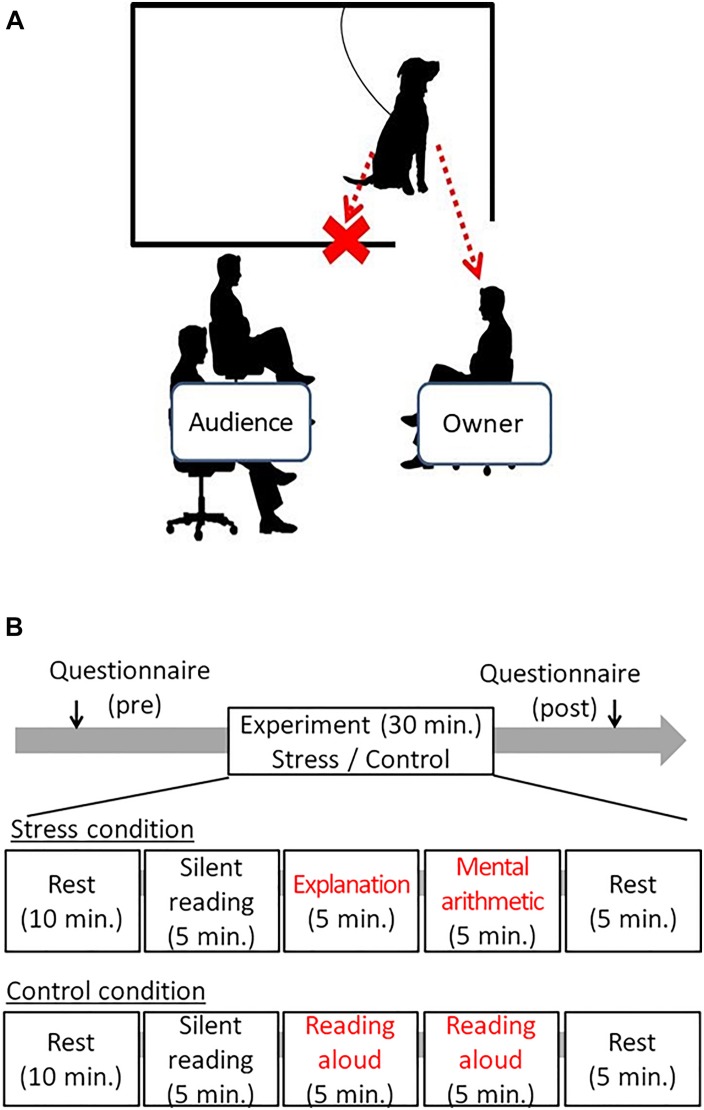
The schematic draw of the experimental setting and the time course of the experiments. **(A)** The owner sat on the chair in front of the audience, and dog only could watch the owner in the sessions. In cases, if the dogs showing separation distress from the owner, the owner sat on the chair close to the dogs. **(B)** In the stress session, 5 min of explanation of the document to the audience, followed by 5 min arithmetic tasks were given to the owner. In the control session, the owner just read the document. The audience was not allowed to give voice communication to the owner in either session.

The experiment took place in an experimental room at Azabu University. An experimental setting is shown in Figure 1A. To inhibit the stimulus given to dogs other than the owner, a partition was placed between the audience and the dog, and the dog could only see the owner. The dog was loosely tied with a leash and could not exit the compartment (1.5 m × 2 m). The owner and the audience sat on chairs and did not walk or stand during the experiment.

### Procedure

The schematic procedure was illustrated in Figure 1B. After informing owners with the experiment contents, the owners who signed the Informed Consent participated in the experiment. The owners were asked to fulfill the attribute data, such as the age of dogs, dog’s sex, status of neutering, and the duration of the ownership. Next, the dog and owner were equipped with electrocardiogram (ECG) devices. Then, the dog and the owner were given 40 min of waiting time in a waiting room together. At that time, the owner was asked to fill out a questionnaire State-Trait Anxiety Inventory (STAI) and to rest (reading books or using the smartphone, etc., was allowed); owners were also instructed not to touch their dogs positively except when spontaneously contacted by their dogs. The owners filled out the STAI again after the test to evaluate the stress levels after the experiment.

After the 40-min resting period, the dog (tied with a loose leash) and the owner entered the experimental room. The experiment was conducted in three steps: (1) filling out the questionnaire pretest and resting time for 40 min, (2) participation in the experimental condition for 30 min, and (3) filling out the post-test questionnaire) (Figure 1B). All dog–owner pairs participated both in Control and Stress conditions on different days, at least 1 week apart. The time of the experiment was fixed to avoid diurnal changes of autonomic activity, and the sequence of two conditions was pseudo-randomly controlled: half of them were given the Control condition first, and the rest were given the Stress condition first.

#### Stress Condition

The Trier social stress test (TSST) is frequently adopted as a method of causing social stress in humans (Buske-Kirschbaum et al., 2003). The standard methods were slightly modified: the original method of 10 min presentation was replaced with a 5-min period to read a document for explanation, and 5-min to explain the document. Subsequently, the participants were asked to conduct verbal mental arithmetic for 5 min. Thus, the stress session consisted of a 5-min speech preparation, 5-min oral explanation of the document, and 5-min verbal mental arithmetic in front of an audience (one experimenter). To minimize social stimulus from the audiences to each owner’s dog, the audiences pointed out an error made by the owner presently participating by presenting a board showing the statement “It’s an error. Repeat the calculation again.”

The control experiment was conducted to exclude possibilities that the utterance itself at the time of the explanation of the document or the math exercise were influencing the dog rather than the owner’s emotional states in front of the audience. The owner was asked merely to read the same documents, but the owner did not need to explanation it. Regarding the control condition of arithmetic, the owners were asked to read loud the formulas and answers written on the paper.

#### Recording Electrocardiograms (ECG) of the Dogs and Their Owners

ECG measurement was conducted according to our previous report (Katayama et al., 2016). The electrocardiogram induction of each dog was an M-X lead. To record the dog’s ECG without shaving, we made a band that combined bandaging tapes (3M Vetrap bandaging tape, 3M, United States) and three disposable electrodes (monitoring electrode 2228, 3M, United States). We parted each dog’s hair so the skin was visually observable about the areas of the manubrium and xiphisternum. Ultrasound gel (Aquasonic clear, Parker, United States) was applied on the surface of the skin. We wrapped three electrodes directly onto each dog’s skin with an elastic bandage. The ECG induction of the owner was a CC5 lead. The owner was asked to fix three electrodes along with the fifth costa. Compact multifunction sensor and myoelectric amplifiers (TSND121 and TS-EMG01, ATR-Promotions, Japan) were used as ECG recording devices. The devices were adjusted for dogs (amplification factor × 250, filter 0.5−150 Hz) and for owners (amplification factor × 1000, filter 0.5–150 Hz). Both ECG sampling rates were set to 1000 Hz.

The ECG data for each dog and owner were recorded on the compact multifunction sensor. At the same time, data for each dog and the data for each owner were sent to a personal computer with a software (SDRecorderT, ATR-Promotions, Japan) in real time to synchronize ECG data with video recorded with cameras (C615 Logicool Co., Ltd.).

#### ECG Analysis

The RRI detection from ECG analysis was the same as in our previous report (Katayama et al., 2016). Briefly, we detected R waves by use of original MATLAB script from recorded ECG data together with visual judgment and calculated RRI. For the HRV analysis, the analysis time-bin was set as 15 continuous seconds; therefore, 40 (*n* = 9) or 41 (*n* = 5) time-bins were obtained in the about 10 min stress/control session. The parameters of the HRV were calculated for each time bin; standard deviation of normal-to-normal R-R intervals (SDNN) that is the index of autonomic nerve systems (ANS), the root mean square of successive heartbeat interval differences (RMSSD) that is the index of parasympathetic nerve system, and mean R-R intervals (mean RRI). If the RRI values were not detected due to mechanical errors for more than 10% of occurrences in each time-bin, the time-bin data were excluded from the analysis.

We excluded 20 out of 34 data from the further analysis. The reasons for exclusion were as follows. (1) Only one condition could be done. (2) The data was not complete for 2 conditions because of the serious video or ECG artifact was happened under either condition. (3) Certain arrhythmias (Second degree atrioventricular block (2nd degree AV block) was found frequently in one dog. 2nd degree AV block can be happened not only the dog with heart disease but also the healthy dogs with rest. However, it was unclear which was the cause so that the dog-owner’s data was excluded.

### Statistical Analysis

For condition comparisons of STAI scores, dog behaviors, and HRV parameters, a Kruskal–Wallis test followed by a Wilcoxson’s signed rank test was conducted. We analyzed these data using statistical software (SPSS ver.22, IBM Co/ Ltd., Japan). In addition, to test the hypothesis that sharing the same environment and bonding can increase the efficacy of emotional contagion, expressed as correlation coefficients of HRV parameters between dogs and owners, the following factors were tested; the duration of the ownership, daily duration of time spent with dogs, age of the dogs, dog’s sex, dog’s body weight, dog’s gazing time to the owner, owner’s gaze time at the dog, touching, by Spearman’s rank correlation coefficient (original MATLAB script), with Benjamini and Hochberg method for false discovery rate of multiple comparison. In these correlation analyses, because the duration of the ownership and dog’s age was highly correlated (*r* = 0.991), and only the duration of the ownership was used for the statistics to avoid multicollinearity. Finally, the effects of these parameters on the correlation coefficients of HRVs were examined by generalized linear model (GLM).

## Results

### Behavior and STAI

The behavior of the dogs in each condition was shown in Table 1. Gazing by a dog to the owner in a stress condition was longer as compared to the control condition (*z* = −2.23, *p* = 0.026, Cohen’s dz = 1.330). Other behavior was not different between the conditions. STAI scores for state anxiety tended to be higher in the stress condition than in the control condition (Supplementary Figure S1, *z* = −1.871, *p* = 0.061, Cohen’s dz = 2.220). Other STAI scores were not different between conditions.

**TABLE 1:** Behaviors of dogs in control and stress conditions.

	**Control**		**Stress**	
**Behavior**	**median**	**quartile(+/−)**	**median**	**quartile(+/-)**
Lie down	90.52	97.3/72.0	82.30	99.1/52.1
Lie	1.62	5.27/0.17	2.32	22.5/0.25
Sit	0.07	0.45/0.00	0.00	2.30/0.00
Stand	2.19	6.68/0.33	0.38	8.35/0.00
Walk	1.05	3.73/0.01	0.48	2.33/0.00
Gazing to the owner	0.66	2.31/0.00	2.34^*^	8.06/0.14
Vocalization (whine)	0.00	0.34/0.00	0.00	0.63/0.00
Vocalization (bark)	0.00	0.00/0.00	0.00	0.00/0.00
Panting	0.00	0.79/0.00	0.00	8.88/0.00
Yawn	0.00	0.35/0.00	0.06	0.33/0.00

Lie down, lying down on the floor with head-down; Lie, lying down on the floor with head-up; Sit, Sitting on the floor; Stand, Stand on the floor; Walk, moving legs for walking; Gazing to the owner, the head and eyes are directed to the owner’ face; Vocalization (whine), whining; Vocalization (bark), barking/woofing; Panting, breathing with panting; Yawn, Yawning. Values in second. Bold characters indicates the significant difference between Control and Stress conditions (^*^p = 0.0122, Wilcoxson’s signed rank test).

### Condition Comparison of HRV

**FIGURE 2:**
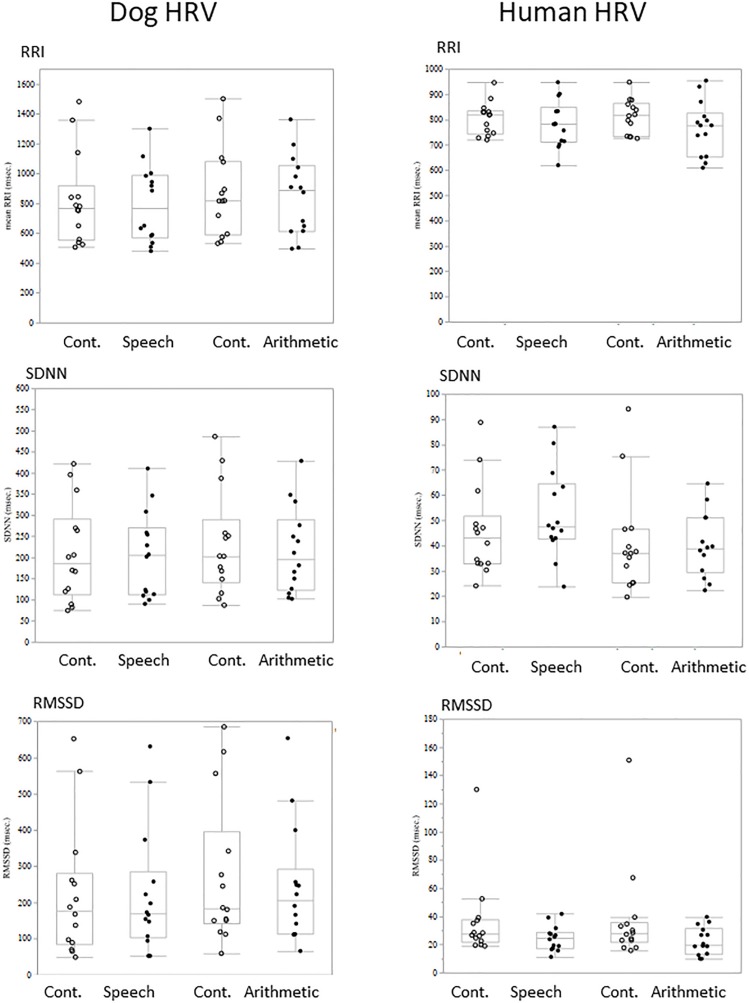
The comparison of HRV parameters between sessions. RRI, SDNN, and RMSSED were calculated in each 5-min session and the values were compared between stress and control sessions. Neither the HRV parameters of Dogs (left panels) and Human (right panels) showed session differences.

Comparisons of each HRV parameter between conditions did not show any significant difference either in dogs or owners (Figure 2).

### Correlation Coefficient Between Dogs and Owners’ HRV

**FIGURE 3:**
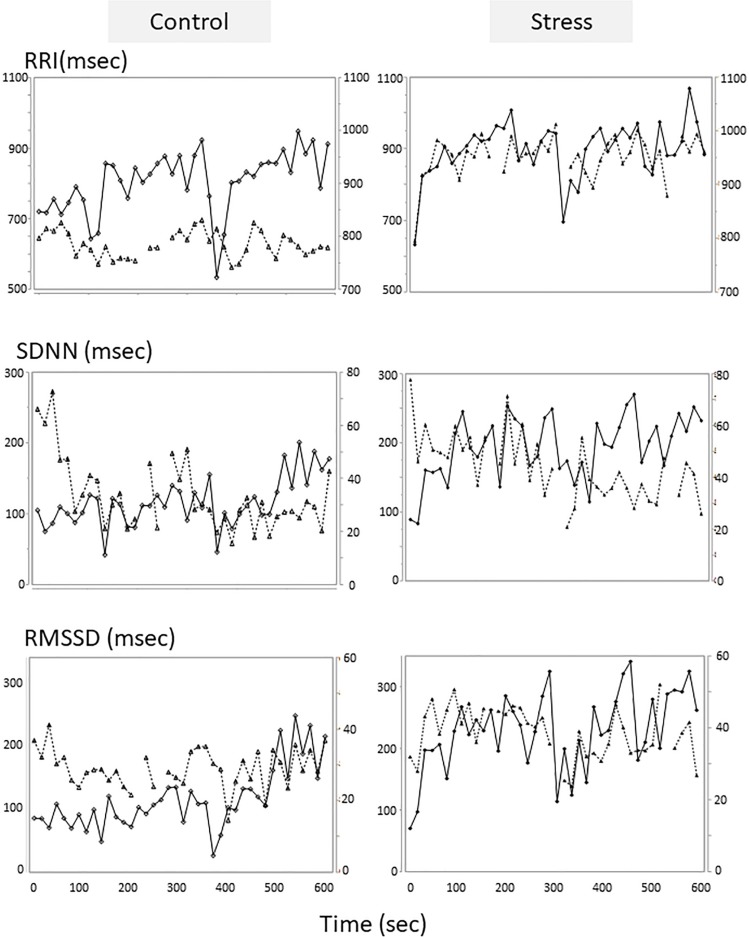
Tentative examples of the HRV parameters changes of a dog and its owner in the sessions. The HRV parameters of the control session were on the left, and those of the stress sessions were on the right panels. The parameters of the dogs were on the first *Y* axis and presented in solid lines with diamond, and the parameters of the owners were on the second *Y* axis and presented in dotted lines with triangle. HRV parameters were calculated in each 15-s time bin.

**FIGURE 4:**
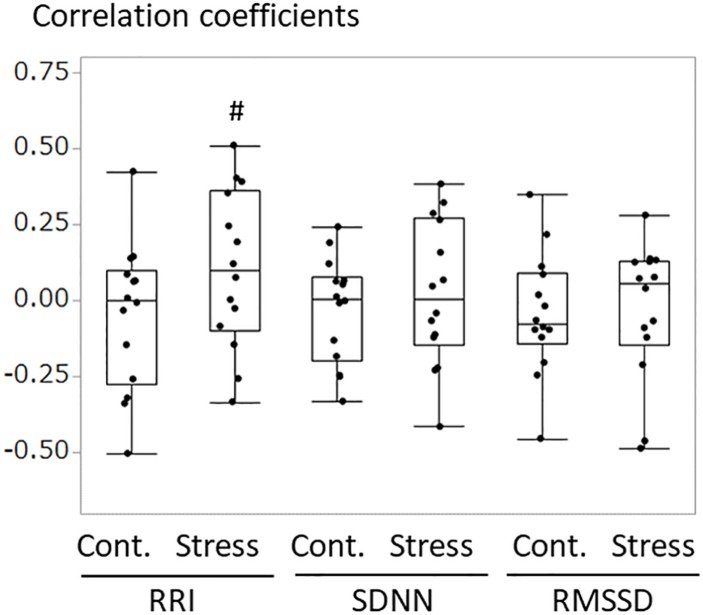
The comparisons of correlation coefficients of RRI, SDNN, and RMSSD between the dog and the owner in each pair. The correlation coefficients were calculated in each pair for 10 min of test/control session. Correlation coefficients in the stress condition tended to be higher as compared to those in the control session, in RRI (*z* = –1.91, ^#^*p* = 0.055, Cohen’s dz = 1.9862).

**FIGURE 5:**
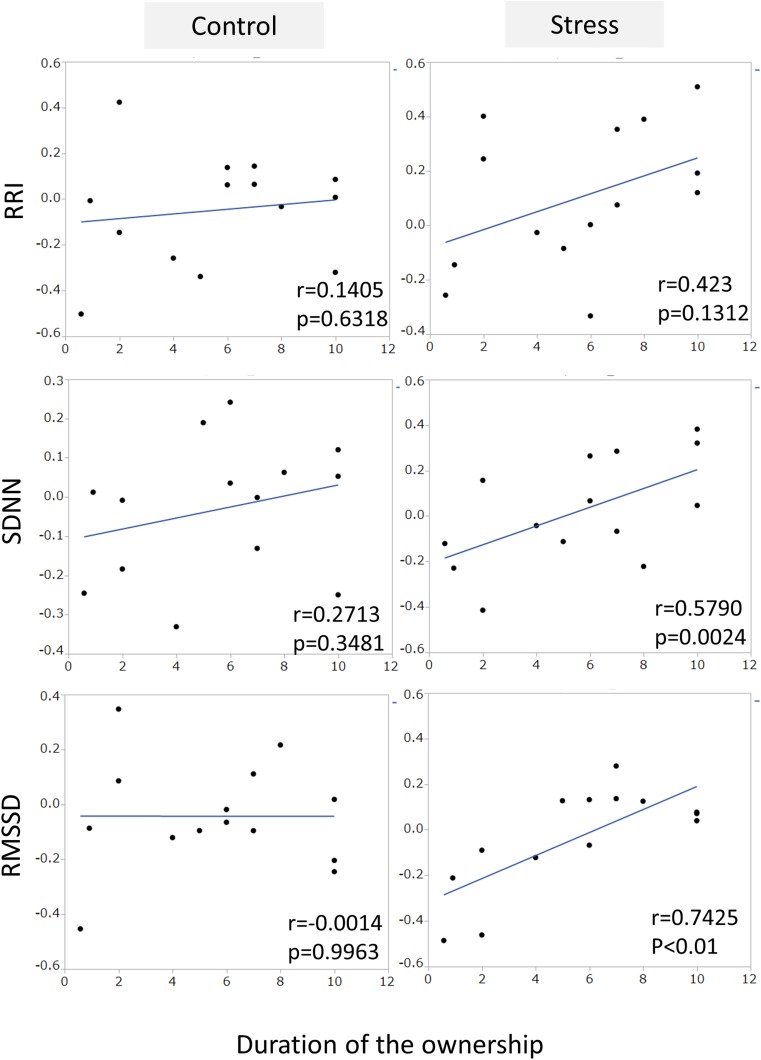
The correlations between the correlation coefficients of RRI, SDNN, and RMSSD in the pairs of dogs and owners, and the duration of ownership. The duration of the ownership had a positive correlation with the correlation coefficient of RMSSD in stress condition, but not in the control condition.

An example of the changes of HRV parameters during the session was shown in Figure 3. Some pairs showed similar time-dependent changes of HRV. Therefore, the correlation coefficients between dogs’ and owners’ HRV were calculated in each pair. Correlation coefficients of RRI in the stress condition tended to be higher than that of the control condition (Figure 4. *z* = −1.91, *p* = 0.055, Cohen’s dz = 1.9862). When the correlation coefficients of HRV parameters between dogs and owners were compared with the other variables (the duration of the ownership, daily duration of time spent with dogs, age of the dogs, dog’s sex, dog’s body weight, dog’s gazing time to the owner, owner’s gaze time at the dog, and touching), there was a significant positive correlations found between the duration of the ownership with the correlation coefficients of RMSSD in the stress condition (Figure 5, RMSSD; *r* = 0.7425, *p* < 0.01). GLM revealed that 5 out of 6 correlation coefficients of HRV parameters were positively influenced by the duration of the ownership (Table 2. Control Condition: RRI, β= 0.033, *p* = 0.026; SDNN, β= 0.027, *p* = 0.001; RMSSD, β= 0.028, *p* = 0.041. Stress Condition: SDNN, β= 0.029, *p* = 0.022; RMSSD, β= 0.072, *p* < 0.001). In addition, daily duration of time spent with dogs also had two positive influences (but one negative) on the correlation coefficients of HRV parameters (Table 2. Control Condition: RRI, β= 0.121, *p* = 0.009; SDNN, β= −0.064, *p* = 0.007. Stress Condition: SDNN, β= 0.121, *p* = 0.001). In the control condition, GLM revealed the effect of sex on correlation coefficients of all three HRV parameters; female dogs showed stronger correlation coefficients as compared to male (Table 2 and Supplementary Figure S2. RRI, β= 0.314, *p* = 0.005; SDNN, β= 0.148, *p* = 0.008; RMSSD, β= 0.229, *p* = 0.020). Owner’s gaze time at the dog and dog’s gaze time at the owner had negative influences on the correlation coefficients of HRV parameters (Table 2).

**TABLE 2:** Results of GLM analysis of correlation coefficients of dog and owners’ HRV.

	**Control**						**Stress**					
	
	**RRI**		**SDNN**		**RMSSD**		**RRI**		**SDNN**		**RMSSD**	
	
**Variables**	**β**	**P**	**β**	**P**	**β**	**P**	**β**	**P**	**β**	**P**	**β**	**P**
Duration of the ownership	**0.033**	**0**.**026**	**0.027**	**0**.**001**	**0.028**	**0**.**041**	0.023	0.308	**0.029**	**0**.**022**	**0.072**	<**0**.**0001**
Daily duration of time spent with dogs	**0.121**	**0**.**009**	**−−0.064**	**0**.**007**	0.054	0.165	–0.051	0.393	**0.121**	**0**.**001**	0.066	0.054
Dog’s sex (M:1, F:2)	**0.314**	**0**.**005**	**0.148**	**0**.**008**	**0.229**	**0**.**020**	–0.134	0.364	0.116	0.139	0.087	0.276
Dog’s body weight	0.000	0.954	**0.008**	**0**.**050**	0.014	0.052	0.008	0.506	**−−0.022**	**0**.**003**	0.002	0.727
Dog’s gazing time to the owner	–0.015	0.459	**−−0.030**	**0**.**010**	0.006	0.746	–0.001	0.836	0.002	0.335	**−−0.004**	**0**.**048**
Owner’s gaze time to the dog	**−−0.668**	**0**.**009**	**−−0.287**	**0**.**024**	**−−0.496**	**0**.**030**	–0.018	0.604	0.003	0.869	–0.003	0.879
Touching	0.065	0.402	**0.110**	**0**.**012**	–0.060	0.404	0.021	0.110	0.002	0.811	–0.017	0.027

*The duration of the ownership, daily duration of time spent with dogs, age of the dogs, dog’s sex, (M, male and assigned as 1. F, female and assigned as 2) dog’s body weight were attribute data from the participating dogs, and owners. Dog’s gazing time to the owner, owner’s gaze time to the dog, touching were behavioral responses observed in the Control/Stress sessions. Bold characters indicates the significant influences on the correlation of coefficients of dog and owner’s HRV parameters.*

## Discussion

In this study, we examined the emotional contagion between humans and dogs by accessing the autonomic responses. It was found that emotional contagion can be transferred from the owner to the dogs, and the efficacy of the emotional contagion was depending of the duration of the time sharing with dog and owners. The existence of emotional contagion between human and dogs, an inter-species dyad, can contribute to the cohabitation.

Previous studies of emotional contagion between dogs and humans evaluated behavioral or hormonal changes (Jones and Josephs, 2006; Romero et al., 2013; Yong and Ruffman, 2014), but these methods contained some concerns. In behavioral parameters, the physiological/neural mechanisms underlying the behaviors cannot be evaluated. Therefore, there was still a concern whether the behavioral changes were results of specific emotional changes or not. For example, emotional changes such as fear can be expressed by various behaviors with large individual differences, but the autonomic fear responses were similar among individuals (Ogata et al., 2006). Therefore, the physiological/neural “emotion” measurement was preferable. Second, the hormonal changes took a relatively longer time, such as minutes to hours, to occur. Instead, emotional changes were more rapid, taking such as 5 to 20 s. Therefore, the higher time-resolution and neuro-physiological measurements are needed to address the question of the existence of emotional contagions between dogs and humans. For these reasons, we focused on HRV. Measurement of HRV has some advantages. For example, HRV reflects the state of the sympathetic and parasympathetic nervous systems in a short time period (Von Borell et al., 2007; Kreibig, 2010). Moreover, HRV parameters are associated with emotion both in humans and animals. The present study confirmed previous studies in dogs in demonstrating an association between the HRV and emotional responses such as affiliation (Romero et al., 2015) and anxiety (Wormald et al., 2017), as well as positive/negative emotions (Katayama et al., 2016). In the present study, the analysis time window was set as 15 sec, which was suitable for accessing the autonomic changes and for accessing the correlation of HRV between humans and dogs. Our results found that some pairs of owners and dogs showed positive correlation of HRV changes in the control and stress sessions. This is the first study, as far as we know, showing correlations of a short-time window HRV between dogs and their owners.

In the stress condition, STAI State anxiety scores tended to be higher than that of the control condition. However, we could not find the effects of stress in human HRV. This suggests that the stress experience by our modified TSST was moderate, even the protocol was the similar to previously described ones. One possible reason for this is that existence of the dog brought social buffering effects to the owners. In humans, the existence of the dog was reported to decrease stress responses, such as autonomic responses (Allen et al., 1991). Because we did not conduct “non-dog” condition, this possibility would be resolved in future. Although the reasons were not clear, some participants showed no change in scores of state anxiety before and after the stress session, indicating that some owners were not stressed. Therefore, the group comparison would not suitable for the evaluation of emotional contagion due to the lack of emotional changes in some owners, and it was needed to access the individual correlation coefficients for the efficacy of the emotional contagion. As a result, correlation coefficients, especially RRI, increased in the stress condition as compared to the control condition. This suggests that when the owners had higher negative emotions, the dogs had similar emotional changes, and it supports previous studies in which emotional contagion is well observed in negative conditions (de Waal, 2009).

One of the most notable findings was that the correlation coefficients of HRV were positively correlated with duration of ownership in both control and stress conditions. In addition, daily duration of the time spent with dogs has positive influence on the correlation coefficients of HRV between dogs and owners. In this experiment, we tested Ohtsuki’s hypothesis that emotional contagion has theoretically evolved by sharing the environment, whereas genetic relatedness is not a large factor (Nakahashi and Ohtsuki, 2018). The present results fully support this hypothesis; the dogs and their owners, as stated above, are genetically distant, but there was emotional contagion; moreover, the sharing environment was a key factor in facilitating emotional contagion. In group living animals, copying each other’s emotion is more adaptive than reacting independently when the environments between individuals are similar (Nakahashi and Ohtsuki, 2018). For example, emotional contagion may help group members to escape from predators, to find food resources, and even to fight against outgroup individuals, all of which are frequently proposed merits of group living (Preston and de Waal, 2002). In Ohtsuki’s theoretical model, once emotional contagion evolves, it works in the same manner between relatives and between non-relatives (Nakahashi and Ohtsuki, 2018). Therefore, the present results constitute the first empirical data for supporting their theoretical model.

The limitation of this study was the small number of sample size (*n* = 14). We have started this experiment of 34 pairs of the owner and dog. However, due to the technical, especially instability of continuous monitoring of HR, 20 pairs were excluded from the analysis. As responding to the owner’s request, we did not shave the hair of the dogs, and this caused the electrical insulation on the pads. More sophisticated methods are needed in future. Even the small sample size, several interesting findings were observed; duration of the ownership were correlated with the correlation coefficient of HRVs between owner and dogs, especially in RMSSD in the stress condition (*p* < 0.001). RMSSD mainly reflects the activity of the parasympathetic nerve, thus sharing the same environment (duration of ownership) can increased the synchronicity of parasympathetic nerve activity between the owner and dogs under the owner’s stress condition. Another interesting point was that, in the control condition, female dogs showed stronger correlation coefficients of HRV parameters with the owners as compared to male. Females animals, including humans, show higher capability in empathy (de Waal, 2009; Ben-Ami Bartal et al., 2011), and this study was consistent with these previous reports. The mechanisms underlying the sex differences in the capability of empathy were not discovered yet, one candidate molecule is oxytocin, which showed sex differences in response to administration in dogs (Nagasawa et al., 2015), and the function was regulated by sex steroid hormones.

One unsolved question concerns the mechanisms underlying the facilitating effect of sharing environments on the expression of emotional contagion between dogs and owners. Living with humans can bring many social experiences to dogs. For example, dogs can form associative learning between environmental risk/threat and owners’ emotional/behavioral reactions. Therefore, owners’ emotional reactions to the environments could be conditioned stimuli to dogs and dogs can form associative learning, and show the similar emotional reaction by observing the owners’ reactions. The other possibility is that a familiar/affiliative relationship exists. While living together, dogs and their owners can form a bond that can facilitate the emotional contagion. Oxytocin is a candidate molecule for explaining this possibility. We observed that eye-gaze from a dog stimulates oxytocin release in the owner, and owner’s talk and touch also stimulate oxytocin release in a dog (Nagasawa et al., 2015). Therefore, the bonding between dog and owner was facilitated by oxytocin acting on the central nervous system (Young and Wang, 2004). It is well known that increase of oxytocin facilitates the reading of others’ emotions and the emergence of empathetic responses (Guastella et al., 2010). Therefore, longer ownership would lead to a tighter bond between dogs and owners, thereby facilitating the oxytocin-mediated positive loop in the experimental context. In fact, the gaze at the owner had a weak positive correlation with duration of the ownership (data not shown, *r* = 0.485, *p* = 0.07). However, the gazing behavior directed by dogs toward their owners had negative influences to the correlation of coefficients of HRV between dogs and owners. Measuring oxytocin both in dogs and humans is needed to clarify this unsolved issue.

The dog is a unique animal, which is the oldest domesticated species. Dogs have coexisted with humans for more than 35,000 years and are woven into human society as partners who bond with humans (Skoglund et al., 2015; Parker et al., 2017). Dogs have acquired human-like communication skills during the domestication process; therefore, they can understand human gestures and facial expressions and can refer to human information in their decision-making. They have come to live together with humans in a group and share the same environment. In this context, emotional contagion can be beneficial to both dogs and humans, such as when a dog alarms the owner to the approach of a predator. Therefore, the emotional contagion found in this study can be the gift of the long cohabitation history.

## Data Availability

The raw data supporting the conclusions of this manuscript will be made available by the authors, without undue reservation, to any qualified researcher.

## Ethics Statement

All experimental procedures were approved by the Animal Ethics Committee of Azabu University (#180410-1) and the Ethical Committee for Medical and Health Research Involving Human Subjects of Azabu University (#052).

## Author Contributions

MK involved in all steps of the process, and was the primary conductor of the experiments. MK, MN, KM, and TKi designed the study and wrote the manuscript. MK and MN performed the statistical analysis. TKu, TY, KF, KN, and KI developed the Matlab codes for data processing.

## Conflict of Interest Statement

The authors declare that the research was conducted in the absence of any commercial or financial relationships that could be construed as a potential conflict of interest.

## Supplementary Material

FIGURE S1 The changes of STAI scores in the owner, before and after the sessions. State anxiety tended to be higher in stress condition as compared to the control condition (*z* = −1.871, ^#^*p* = 0.061). Other STAI scores were not different between conditions (data not shown).

FIGURE S2 Correlation coefficients of RRI (top), SDNN (middle), and RMSSD (bottom) for male and female in the control condition.

TABLE S1 Dog’s information participated in this study.

## References

[B1] Allen K. M. Blascovich J. Tomaka J. Kelsey R. M. (1991). Presence of human friends and pet dogs as moderators of autonomic responses to stress in women. *J. Pers. Soc. Psychol.* 61 582–589. 10.1037//0022-3514.61.4.582 1960650

[B2] Bartal I. B. Rodgers D. A. Sarria M. S. B. Decety J. Mason P. (2014). Pro-social behavior in rats is modulated by social experience. *eLife* 3:e01385. 10.7554/eLife.01385 24424411 PMC3884117

[B3] Ben-Ami Bartal I. Decety J. Mason P. (2011). Empathy and pro-social behavior in rats. *Science* 334 1427–1430. 10.1126/science.1210789 22158823 PMC3760221

[B4] Brosschot J. F. Thayer J. F. (2003). Heart rate response is longer after negative emotions than after positive emotions. *Int. J. Psychophysiol.* 50 181–187. 10.1016/s0167-8760(03)00146-6 14585487

[B5] Buske-Kirschbaum A. von Auer K. Krieger S. Weis S. Rauh W. Hellhammer D. (2003). Blunted cortisol responses to psychosocial stress in asthmatic children: a general feature of atopic disease? *Psychosom. Med.* 65 806–810. 10.1097/01.psy.0000095916.25975.4f 14508024

[B6] de Waal F. B. (2009). *The Age of Empathy.* New York, NY: Broadway Books.

[B7] Edgar J. L. Lowe J. C. Paul E. S. Nicol C. J. (2011). Avian maternal response to chick distress. *Proc. Biol. Sci.* 278 3129–3134. 10.1098/rspb.2010.2701 21389025 PMC3158930

[B8] Guastella A. J. Einfeld S. L. Gray K. M. Rinehart N. J. Tonge B. J. Lambert T. J. (2010). Intranasal oxytocin improves emotion recognition for youth with autism spectrum disorders. *Biol. Psychiatry* 67 692–694. 10.1016/j.biopsych.2009.09.020 19897177

[B9] Jeon D. Kim S. Chetana M. Jo D. Ruley H. E. Lin S. Y. (2010). Observational fear learning involves affective pain system and Cav1.2 Ca2+ channels in ACC. *Nat. Neurosci.* 13 482–488. 10.1038/nn.2504 20190743 PMC2958925

[B10] Jones A. C. Josephs R. A. (2006). Interspecies hormonal interactions between man and the domestic dog (Canis familiaris). *Horm. Behav.* 50 393–400. 10.1016/j.yhbeh.2006.04.007 16784746

[B11] Katayama M. Kubo T. Mogi K. Ikeda K. Nagasawa M. Kikusui T. (2016). Heart rate variability predicts the emotional state in dogs. *Behav. Process.* 128 108–112. 10.1016/j.beproc.2016.04.015 27129806

[B12] Kreibig S. D. (2010). Autonomic nervous system activity in emotion: a review. *Biol. Psychol.* 84 394–421. 10.1016/j.biopsycho.2010.03.010 20371374

[B13] Langford D. J. Crager S. E. Shehzad Z. Smith S. B. Sotocinal S. G. Levenstadt J. S. (2006). Social modulation of pain as evidence for empathy in mice. *Science* 312 1967–1970. 10.1126/science.1128322 16809545

[B14] Nagasawa M. Mitsui S. En S. Ohtani N. Ohta M. Sakuma Y. (2015). Oxytocin-gaze positive loop and the coevolution of human-dog bonds. *Science* 348 333–336. 10.1126/science.1261022 25883356

[B15] Nagasawa M. Mogi K. Kikusui T. (2009). Attachment between humans and dogs. *Jnp. Psychol. Res.* 51 209–221. 10.1111/j.1468-5884.2009.00402.x

[B16] Nakahashi W. Ohtsuki H. (2018). Evolution of emotional contagion in group-living animals. *J. Theor. Biol.* 440 12–20. 10.1016/j.jtbi.2017.12.015 29253506

[B17] Ogata N. Kikusui T. Takeuchi Y. Mori Y. (2006). Objective measurement of fear-associated learning in dogs. *J. Vet. Behav.* 1 55–61. 10.1016/j.jveb.2006.06.002

[B18] Parker H. G. Dreger D. L. Rimbault M. Davis B. W. Mullen A. B. Carpintero-Ramirez G. (2017). Genomic analyses reveal the influence of geographic origin, migration, and hybridization on modern dog breed development. *Cell Rep.* 19 697–708. 10.1016/j.celrep.2017.03.079 28445722 PMC5492993

[B19] Preston S. D. de Waal F. B. (2002). Empathy: its ultimate and proximate bases. *Behav. Brain Sci.* 25:71. 12625087 10.1017/s0140525x02000018

[B20] Romero T. Konno A. Hasegawa T. (2013). Familiarity bias and physiological responses in contagious yawning by dogs support link to empathy. *PLoS One* 8:e71365. 10.1371/journal.pone.0071365 23951146 PMC3737103

[B21] Romero T. Nagasawa M. Mogi K. Hasegawa T. Kikusui T. (2014). Oxytocin promotes social bonding in dogs. *Proc. Natl. Acad. Sci. U.S.A.* 111 9085–9090. 10.1073/pnas.1322868111 24927552 PMC4078815

[B22] Romero T. Nagasawa M. Mogi K. Hasegawa T. Kikusui T. (2015). Intranasal administration of oxytocin promotes social play in domestic dogs. *Commun. Integr. Biol.* 8:e1017157. 10.1080/19420889.2015.1017157 26478773 PMC4594226

[B23] Skoglund P. Ersmark E. Palkopoulou E. Dalén L. (2015). Ancient wolf genome reveals an early divergence of domestic dog ancestors and admixture into high-latitude breeds. *Curr. Biol.* 25 1515–1519. 10.1016/j.cub.2015.04.019 26004765

[B24] Sümegi Z. Oláh K. Topál J. (2014). Emotional contagion in dogs as measured by change in cognitive task performance. *Appl. Anim. Behav. Sci.* 160 106–115. 10.1016/j.applanim.2014.09.001

[B25] Von Borell E. Langbein J. Després G. Hansen S. Leterrier C. Marchant-Forde J. (2007). Heart rate variability as a measure of autonomic regulation of cardiac activity for assessing stress and welfare in farm animals—a review. *Physiol. Behav.* 92 293–316. 10.1016/j.physbeh.2007.01.007 17320122

[B26] Wormald D. Lawrence A. J. Carter G. Fisher A. D. (2017). Reduced heart rate variability in pet dogs affected by anxiety-related behaviour problems. *Physiol. Behav.* 168 122–127. 10.1016/j.physbeh.2016.11.003 27838312

[B27] Yong M. H. Ruffman T. (2014). Emotional contagion: dogs and humans show a similar physiological response to human infant crying. *Behav. Process.* 108 155–165. 10.1016/j.beproc.2014.10.006 25452080

[B28] Young L. J. Wang Z. (2004). The neurobiology of pair bonding. *Nat. Neurosci.* 7 1048–1054. 10.1038/nn1327 15452576

