# Supplementary figures and images for: Emotional Contagion From Humans to Dogs Is Facilitated by Duration of Ownership
Source: Front Psychol. 2019 Jul 19;10:1678. doi: 10.3389/fpsyg.2019.01678 (PMC6658615; doi:10.3389/fpsyg.2019.01678)

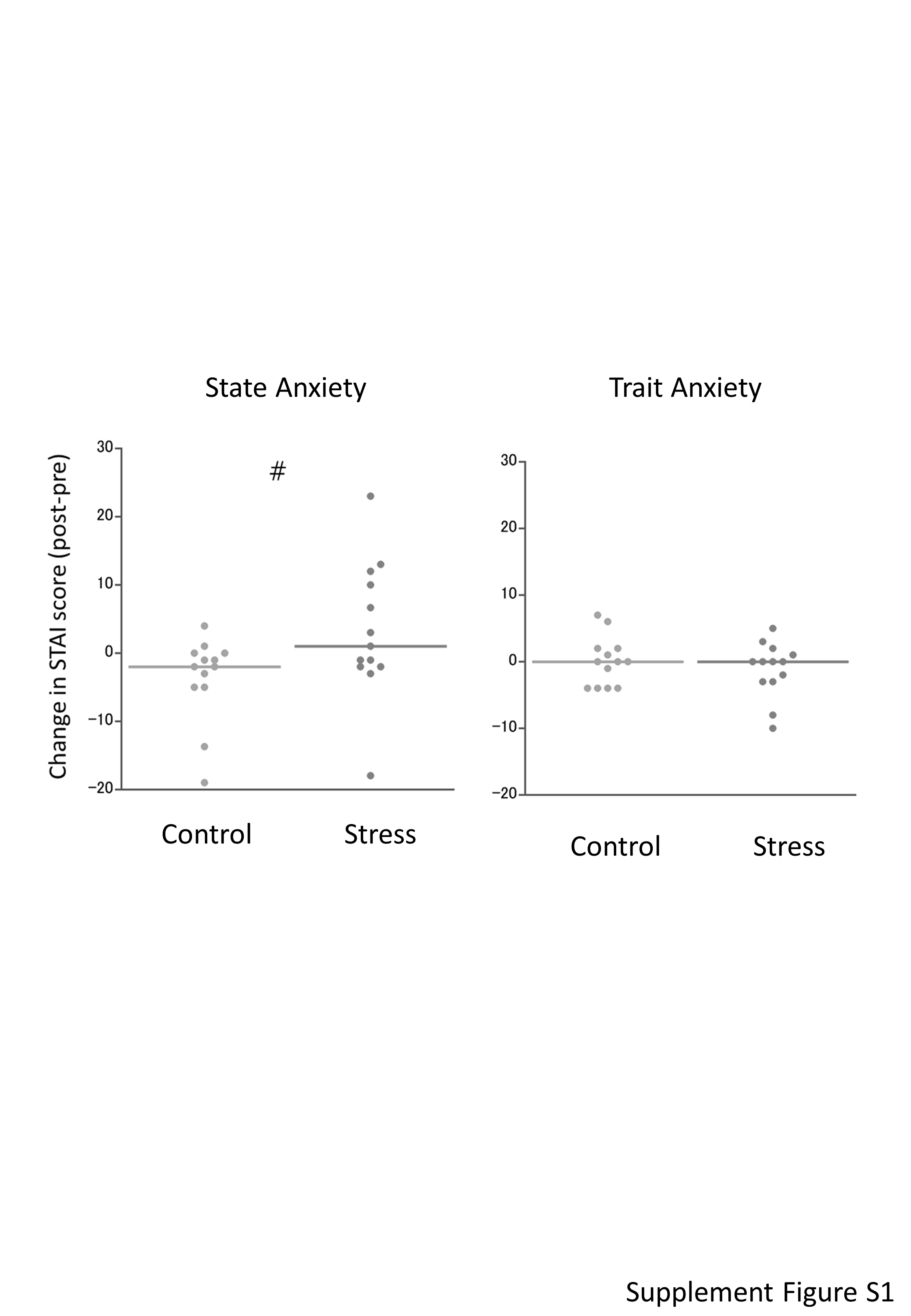

Supplement: FIGURE S1 — The changes of STAI scores in the owner, before and after the sessions. State anxiety tended to be higher in stress condition as compared to the control condition (z = −1.871, #p = 0.061). Other STAI scores were not different between conditions (data not shown). [file Image_2.TIF]

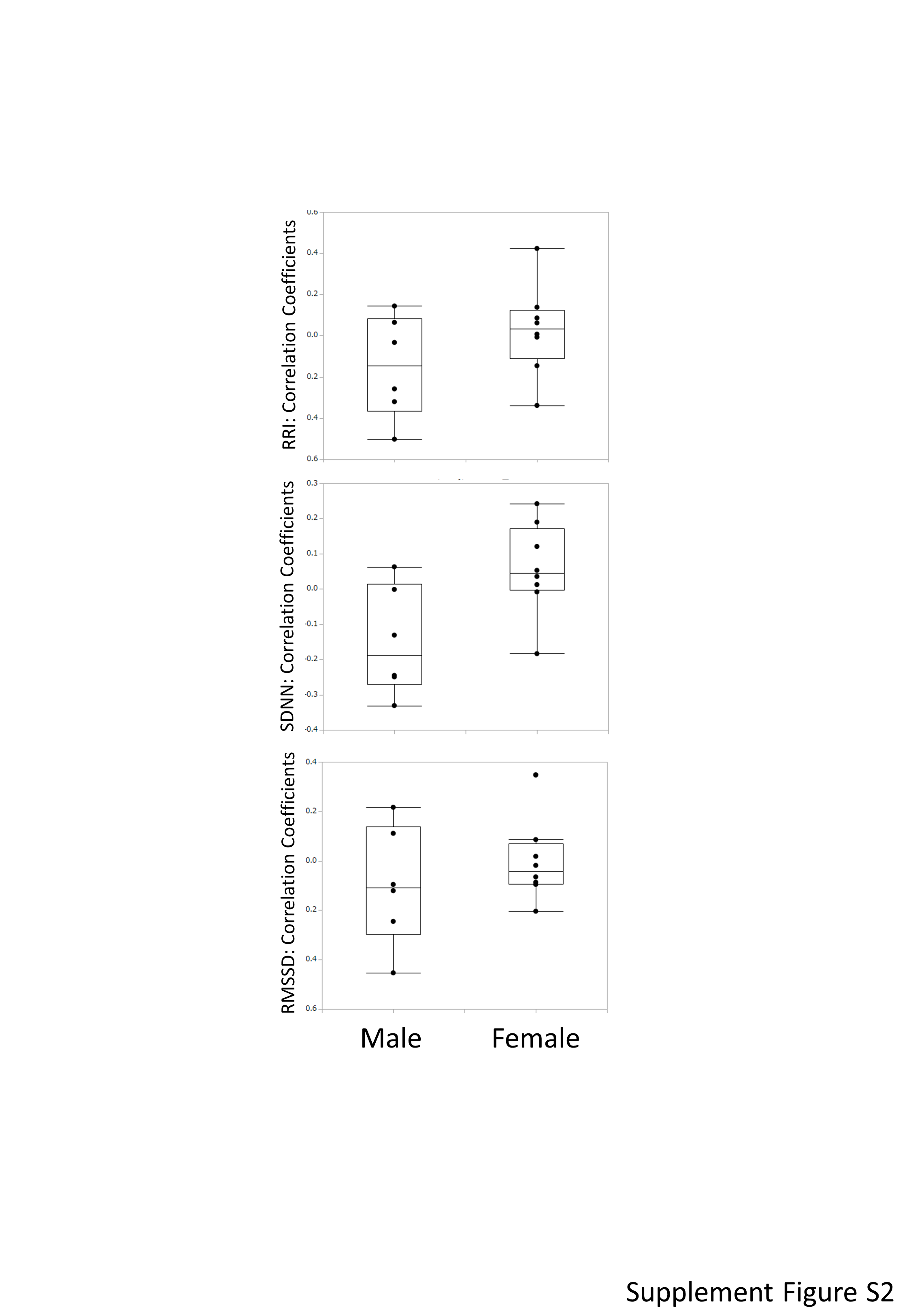

Supplement: FIGURE S2 — Correlation coefficients of RRI (top), SDNN (middle), and RMSSD (bottom) for male and female in the control condition. [file Image_3.TIF]

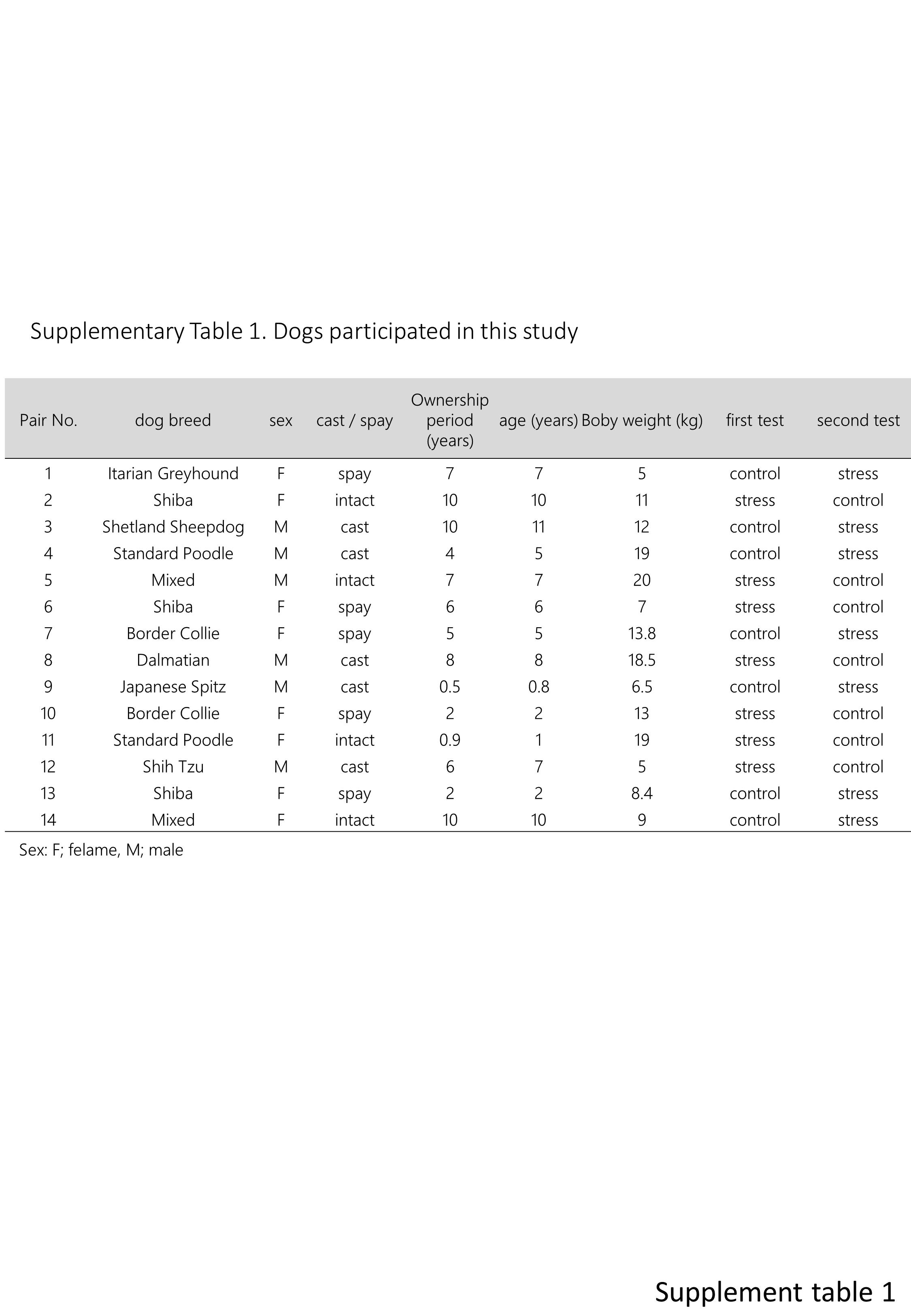

Supplement: TABLE S1 — Dog’s information participated in this study. [file Image_1.TIF]
